# Interpretable machine learning-driven multi-omics risk stratification and drug repurposing nominates Treg/Th17 with gluconeogenesis/lactylation integration as a prognostic and druggable biomarker for glioblastoma patients

**DOI:** 10.3389/fonc.2026.1761182

**Published:** 2026-07-14

**Authors:** Siqi Xie, Weiming Chen, Bing Zhang, Shangeng Weng

**Affiliations:** 1 Department of Hepatopancreatobiliary Surgery, Fujian Abdominal Surgery Research Institute,The First Affiliated Hospital of Fujian Medical University, Fuzhou, Fujian, China; 2 Department of Hepatopancreatobiliary Surgery, National Regional Medical Center, Binhai Campus of the First Affiliated Hospital, Fujian Medical University, Fuzhou, Fujian, China; 3 Fujian Children’s Hospital, Fuzhou, Fujian, China

**Keywords:** glioblastoma, gluconeogenesis/lactylation, ODC1, prognosis, therapeutic approaches, Treg/Th17

## Abstract

**Objective:**

Dysregulation of Treg/Th17 balance and gluconeogenesis/lactylation contributes to glioblastoma (GBM) progression. Hence, it is essential for gaining insights into their mechanisms in GBM.

**Methods:**

By integrating ssGSEA, Limma and WGCNA frameworks and GBM cerebral public bulk profiles from GEO database with gluconeogenesis and lactylation gene list acquired from Genecard database, we identified Treg/Th17 and gluconeogenesis/lactylation (TGL)-associated shared DEGs for GBM patients. Next, Lasso-cox regression analysis pointed out a TGL-associated risk stratification model in TCGA-GBM training cohort and GEO independent validation dataset. Besides, the immune and intratumoral heterogeneity between high-risk and low-risk groups were assessed. Besides, SHAP made Lasso-cox regression analysis interpretable and identification of TGL-associated hub gene. The expression value, and the association of hub gene with TGL and intratumoral features of GBM were further validated *in silico* and *in vitro*. Significantly, the hub gene heterogeneity in GBM single-cell level was also estimated at temporal and spatial manners, especially in artificial intelligence (AI)-driven virtual cells. Finally, ridge regression and molecular docking were performed for identification of optimal therapeutic strategy by targeting hub gene for GBM patients and then validated at *in vitro* studies.

**Results:**

Integrated TGL can guide the risk stratification and prognostic model construction for GBM patients. ODC1 can be considered as up-regulated TGL-related regulator involved in GBM pathogenesis. THZ-2-102–1 can be considered as potential drug targeting ODC1 for the treatment of GBM.

**Conclusion:**

Our study first integrated TGL-associated gene signature in GBM patient risk stratification and therapeutic framework for GBM patients via machine learning and multi-omics, which provides novel ideas into GBM patient clinical translation.

## 1 Introduction

Glioblastoma (GBM) is recognized as the most common and aggressive type of brain tumor, with an alarming incidence rate of approximately 25,000 new cases annually worldwide (1). The prognosis for patients diagnosed with GBM is dire, with a five-year survival rate of less than 5% (2). Current treatment modalities, including surgical resection, radiation therapy, and chemotherapy, have shown limited efficacy, emphasizing the urgent need for novel therapeutic strategies (3, 4).

The molecular mechanisms underlying GBM are complex and multifaceted. For example, dysregulation of gluconeogenesis or glucose metabolism contributes to the progression of GBM (5). Besides, dysregulation of gluconeogenesis and glucose metabolic reprogramming also lead to excessive lactylation in GBM tumor microenvironment(TME), which results in the metastasis and growth of GBM cancer cells (6). Indeed, in the aspect of tumor immune microenvironment, Treg also can shape a tumor suppressive micro-environment for GBM patients (7). A report pointed out dysregulation of glutamate transport can enhance Treg function and facilitate angiogenesis for GBM patients (8). Besides, dysregulation of Treg/Th17 proportion also crystally modulates the progression of various cancer (9). Recent studies also pointed out glucose metabolism plays a key role in the regulation of Treg/Th17 balance (10). Dysregulation of glucose metabolism can lead to the excessive accumulation of lactate, thereby promoting lactylation and expression of oncogenic molecules involved in cancer progression and Treg functions (11). Aberrant lactylation also can affecting Treg/Th17-associated modulator expressions and various malignant progression (12). Specifically, in GBM, oxamate can increase lactate accumulation and regulate Treg infiltration via increasing CD39, CD73 and CCR8 expression in a histone H3K18 lactylation manner (13). However, the integrated mechanisms or mechanisms of gluconeogenesis/lactylation in regulation of Treg/Th17 in GBM pathogenesis have not yet been elucidated.

**Figure 1:**
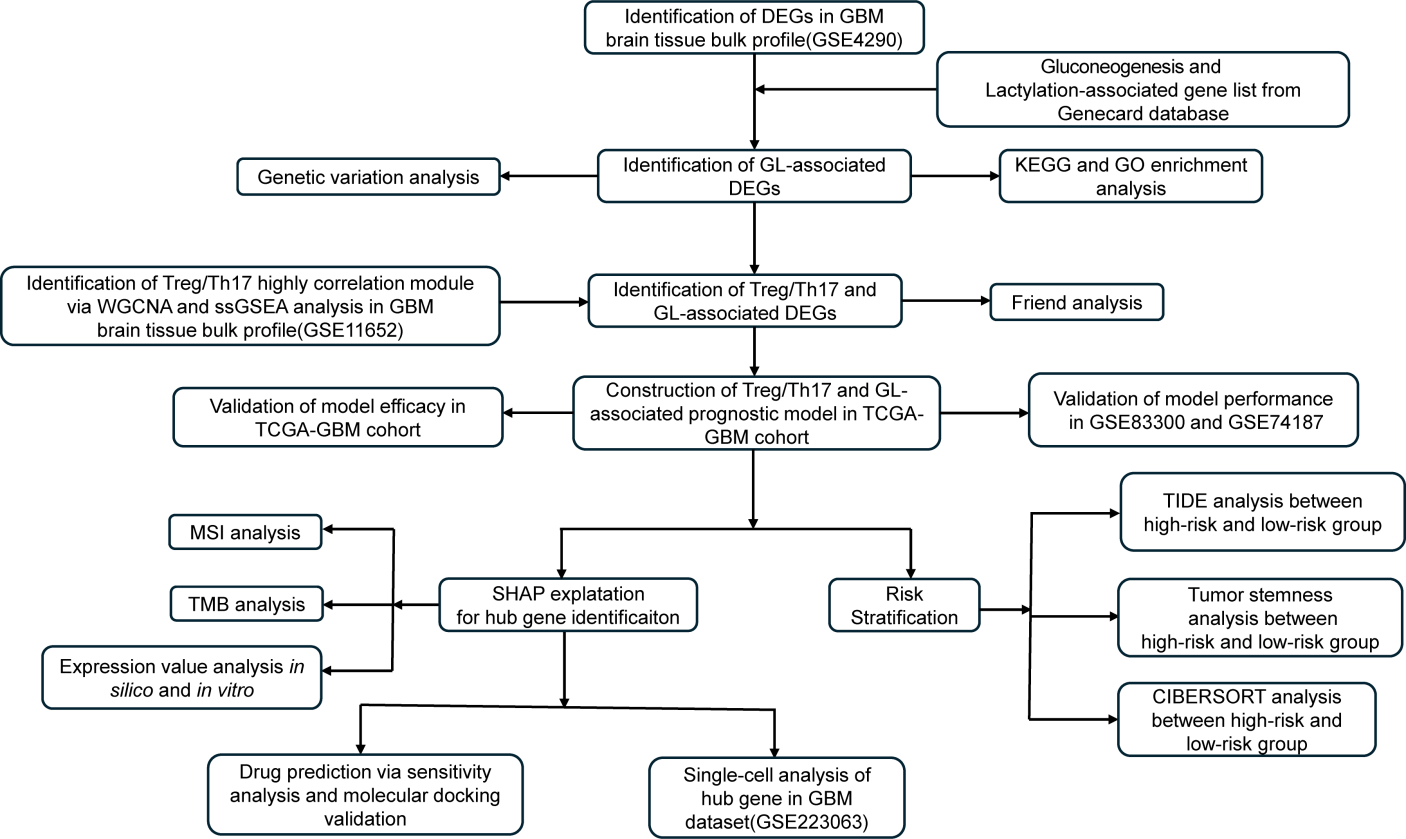
The workflow of this study.

In this study, we creatively integrated Treg/Th17 signature and gluconeogenesis/lactylation signature (TGL) to decipher their co-pathogenic effects on GBM progression via end-to-end machine learning pipelines and multi-omics, providing novel ideas into GBM pathogenesis. Besides, ODC1 also can be considered as TGL-related hub gene involved in regulating GBM pathogenesis and TGL patterns. In addition, THZ-2-102–1 can be considered as potential drug targeting ODC1 for the treatment of GBM. We described the workflow of this study in **Figure 1**.

## 2 Materials and methods

### 2.1 Source of bulk profile

GBM brain tissue bulk profiles(GSE4290, GSE11652, GSE83300 and GSE74187) were acquired from GEO database via GEOquery package of R (14). GSE4290 was based on GPL570, which includes 23 control samples and 76 GBM samples. GSE11652 was based on GPL10558, which includes 8 control samples and 34 GBM samples. GSE8330 was based on GPL6480, which includes 50 GBM samples. GSE74187 was based on GPL6480, which includes 60 GBM samples. Integration GSE83300 and GSE74187 was analyzed by sva package of R for removing batch effects (15). We also downloaded GSE83300 and GSE74187 integration corresponding clinical information. All aforementioned dataset was normalized and standardized via Limma package of R (16). We downloaded STAR-counts data and corresponding clinical information for 174 tumors from the TCGA database. We then extracted data in TPM format and performed normalization using the log2(TPM + 1) transformation via edgeR package of R (17). Gluconeogenesis and lactylation-associated gene lists with Treg/Th17 balance-associated gene list were acquired from Genecards database with a threshold set at >1.

### 2.2 Identification of DEGs

In GSE4290, the criteria for identifying significant differentially expressed genes (DEGs) in the integrated dataset were established with a threshold of | log2FC | > 0.5 and padj < 0.05, utilizing the Limma package in R (16). KEGG and GO enrichment analyses were performed using the ClusterProfiler package in R, based on the hallmark gene set acquired from MSIGDB (18). Genetic variation was analyzed by maftools package of R (19). Gluconeogenesis and lactylation-associated gene lists were intersected with DEGs in GSE4290 for identification of gluconeogenesis and lactylation-associated DEGs.

### 2.3 WGCNA analysis

ssGSEA utilizes a deconvolution algorithm to evaluate the composition and abundance of different immune cell types within a heterogeneous cellular mixture, relying on transcriptomic data as its basis (20). In this investigation, we initially examined the proportions of 22 distinct immune cell types within both normal and GBM samples obtained from the GSE11652 dataset. To identify gene modules that demonstrate a high degree of correlation, weighted gene co-expression network analysis (WGCNA) was conducted. This analysis aimed to clarify the interrelationships among these modules and evaluate their associations with external sample characteristics, ultimately seeking to uncover potential biomarkers or therapeutic targets (21). In our study, WGCNA was executed using the R package WGCNA to identify modules that exhibited the strongest correlation with GBM and Treg/Th17 in patients diagnosed with GBM (22). Initially, we processed the sample data to eliminate any outliers. Following this, a correlation matrix was generated using the WGCNA software package of R (23). The optimal soft threshold was determined to convert the correlation matrix into an adjacency matrix, from which a topological overlap matrix (TOM) was subsequently derived. The TOM-based phase dissimilarity metric was employed to cluster genes with analogous expression profiles into gene modules through average linkage hierarchical clustering. Module was identified that exhibited the highest correlation with Treg/Th17 was selected. Finally, DEGs associated with the gluconeogenesis and lactylation were intersected with the Treg/Th17 high-correlated module for identification of TGL-associated shared DEGs. Besides, the KEGG and GO enrichment analysis was performed for investigation of molecular and biological functions of TGL-associated DEGs via the ClusterProfiler package in R in accordance with KEGG and GO gene set sourced from the MSIGDB database (18).

### 2.4 Interpretable Lasso-cox regression analysis

In order to establish TGL-associated predictive models for prognosis and to identify key variables related to GBM, we conducted Lasso-Cox regression and SHAP analysis utilizing the TCGA-GBM cohort, followed by validation in the GSE83300 and GSE74187 integrating datasets. The configuration of the Lasso-Cox algorithm was assessed using the area under the curve (AUC) as a performance metric. To further develop a prognostic model for TGL, we integrated samples along with corresponding clinical data from both the TCGA-GBM and integrated GSE83300 and GSE74187 datasets, employing Kaplan-Meier (KM) analysis and time-dependent receiver operating characteristic (ROC) analysis. Notably, the variables contributing to the construction of the Lasso-Cox regression model were examined using the SHAP model (24). Additionally, we evaluated the model performance via nomogram, calibration, and DCA analysis. Next, CIBERSORT analysis was performed for investigation of immune cell proportion between TGL-associated high risk and low risk groups distinguished by TGL-associated prognostic model in TCGA-GBM cohort (25). Besides, tumor stemness between TGL-associated high risk and low risk groups was also assessed by OCLR package of R in TCGA-GBM cohort (26). Response to immunotherapy between TGL-associated high risk and low risk groups was assessed by TIDE algorithm of R in TCGA-GBM cohort (27). Next, the most important contributor analyzed by SHAP analysis was considered as TGL-associated hub gene, and hub gene molecular and immune features in TCGA-GBM cohort via ESTIMATE package of R and single-gene GSEA analysis powered by the ClusterProfiler package in R in accordance with KEGG and GO gene set sourced from the MSIGDB database (18, 28). Besides. The expression of ODC1 in TCGA-GBM cohort, and association of ODC1 with TMB and MSI in TCGA-GBM cohort was estimated by ggplot2 and ggstatsplot packages of R (29, 30). Besides, co-expression patterns between hub gene and gluconeogenesis, lactylation and Treg/Th17 balance key regulators were also assessed in TCGA-GBM cohort.

### 2.5 Single-cell transcriptomic analysis

Initially, we acquired the single-cell transcriptomic dataset related to GBM (GSE223063) from the Gene Expression Omnibus (GEO) database. The analysis of the single-cell RNA sequencing (scRNA-seq) data involved several essential steps, including quality control (QC), dimensionality reduction, and marker identification, all conducted using the Seurat R package (31). QC was rigorously implemented for each individual cell based on established criteria: gene counts were restricted to a range between 200 and 6000, unique molecular identifier (UMI) counts needed to surpass 1000, and the proportion of mitochondrial genes was limited to below 10%. Following the QC procedures, the dataset was normalized, enabling the identification of 2000 genes exhibiting significant variability for subsequent analyses. After normalization, dimensionality reduction techniques, specifically t-SNE and UMAP, were applied. Cell type annotations were executed utilizing the scMayoMap algorithm within R software (32). The expression levels of the target genes were assessed across the various annotated cell populations. Intercellular communication networks were inferred through the application of the CellChat package in R (33). Furthermore, we examined the energy metabolic pathways at the single-cell level among the annotated cell populations by utilizing the energy Metabolism package in R (34). We also performed single-cell gene set enrichment analysis (ssGSEA) to investigate the functional enrichment of hub genes at a single-cell resolution, using the hallmark gene set sourced from the MSIGDB database via the ClusterProfiler package in R (35).Pseudo-time analysis of targeted gene expressions within specific cell types was conducted using the monocle2 package in R (36). Virtual cell knockout of targeted gene in targeted cell type(interneuron annotated at single-cell level) was performed by scTenifoldKnk package of R (37).

### 2.6 Drug prediction and molecular docking

A thorough methodology integrating network pharmacology with molecular docking techniques was utilized to systematically investigate the potential interactions between pharmaceutical compounds and their respective targets. The prediction process was executed using GSCA database and the R package pRRophetic (38). The estimation of the half-maximal inhibitory concentration (IC50) for the samples was conducted via ridge regression analysis. Molecular docking analyses were carried out to evaluate the interactions between the drugs and the target proteins. The Protein Data Bank (PDB) files corresponding to the target proteins were obtained from the RCSB PDB, while ligand structures were extracted in SDF format from the PubChem database. Following this, molecular docking was performed to assess the binding affinities between the selected proteins and the compounds of interest. Initially, PyMOL software (Version 2.6.0) was employed to remove water molecules and ligands, retaining solely the protein backbone. Subsequently, the AutoDock Vina Tool (Version 4.2.6) was utilized to identify potential binding sites on the protein surface and to perform flexible molecular docking. This process entailed calculating docking scores and binding affinities (expressed as Vina scores in kcal/mol) for each identified binding site. The five most favorable binding sites were ranked based on their binding energies, with the site demonstrating the lowest binding energy chosen for visualization in PyMOL. This visualization underscored the locations of hydrogen bonds linked to ligand interactions within the resulting imagery. The findings were then illustrated in PyMOL to represent the binding modes and hydrogen bonding interactions effectively.

### 2.7 Cell lines and culture

The cell lines C6(rat GBM cancer cell line), HT-22(mouse hippocampal neural cell line), U87(human GBM cancer cell line) and HEB(human astrocyte neural cell line) were obtained from the Shanghai Academy of Biological Sciences located in Shanghai, China. The U87, HEB, C6 and HT-22 cell lines were grown in Roswell Park Memorial Institute (RPMI) 1640 complete medium, which was supplemented with a 1% antibiotic solution comprising both penicillin and streptomycin, along with 10% fetal bovine serum (FBS, Gibco). All cell passage procedures were conducted under strictly controlled conditions of 37 °C and 5% CO2 within a humidified incubator. In contrast, the HEK293T cell line, procured from the Shanghai Academy of Biological Sciences located in Shanghai, China, was cultivated in Dulbecco’s Modified Eagle Medium (DMEM), which was also supplemented with a 1% penicillin-streptomycin solution and 10% FBS. All procedures for cell passage were conducted under strictly controlled conditions of 37 °C and 5% CO2 within a humidified incubator.

### 2.8 q-RT-PCR

Total RNA was isolated utilizing TRIzol reagent (TaKaRa, Beijing, China), with its concentration, purity, and integrity assessed via a NanoDrop spectrophotometer (Thermo Scientific, Waltham, MA, USA). For the reverse transcription procedure, 1 µg of total RNA was mixed with HiScript II Q RT SuperMix for qPCR (+gDNA wiper), in conjunction with a gDNA eraser (Vazyme, Shanghai, China). Following this, the concentration, purity, and integrity of the resultant cDNA were evaluated using the same NanoDrop spectrophotometer. The quantitative reverse transcription polymerase chain reaction (qRT-PCR) was performed with SYBR Green MasterMix (11203ES50, YEASEN, Shanghai, China) and StepOne Software v.2.3 (Applied Biosystems, Carlsbad, CA, USA) over 40 amplification cycles, ensuring three biological replicates for each sample. Data analysis was executed using the ΔΔCt (cycle threshold) method, normalizing the results against the expression levels of the reference gene, GAPDH. The primer sequences utilized in the qRT-PCR assays are provided below:

For U87 and HEB,

ODC1:

F:5′‐TTTACTGCCAAGGACATTCTGG‐3′
R:5′‐GGAGAGCTTTTAACCACCTCAG‐3′

GAPDH:

F:5′‐ GAGAAGGCTGGGGCTCATTT‐3′
R 5′‐ ATGACGAACATGGGGGCATC‐3′.

For C6 and HT-22,

ODC1:

F:5'- AGGCCACACTGGCAACTCA-3'
R:5′‐TGCGCTCAGTTCTGGTACTTCA‐3′

GAPDH:

F:5 '-ACCCACTCCTCCACCTTTGAC-3'
R:5 '-TGTTGCTGTAGCCAAATTCGTT-3'

### 2.9 Silencing via shRNA

The shRNA sequence designed for ODC1 knockdown was:

GCCATATGGAAGACTAGGATA

This sequence was cloned into the pLKO.1 lentiviral plasmid vector. The shRNA-encoding plasmids were co-transfected with the VSV-G envelope plasmid and the psPAX packaging plasmid into HEK293T cells using Lipofectamine 2000 (Thermo Fisher Scientific) according to the manufacturer’s protocol. The following day, the culture medium was refreshed. Three days of post-transfection, the lentivirus-containing supernatants were collected, filtered, and used to infect target cells in the presence of 4 μg/ml polybrene (Sigma-Aldrich). U87 cells were plated in 24-well plates at a density of 5×10^4 cells per well and cultured until they reached 50–70% confluence. The standard medium was then replaced with a diluted sh-ODC1 lentiviral solution for infection. After 72 hours of incubation, the cells were trypsinized, washed with phosphate-buffered saline (PBS), and seeded into 10 cm culture dishes at a density of 500 cells per dish. Puromycin (Thermo Scientific) selection was applied for three weeks. Surviving clones were identified by the formation of cloning rings, subsequently expanded, and subcloned using the limiting dilution method.

### 2.10 Western blotting

Following the application of various treatments, the cells were thoroughly washed with ice-cold phosphate-buffered saline (PBS) obtained from Hyclone in Seattle, WA, USA, and were subsequently collected through gentle scraping. The total protein extraction was performed by lysing the cells with radioimmunoprecipitation assay (RIPA) lysis buffer supplied by Beyotime, Shanghai, China, which was supplemented with a combination of phosphatase and protease inhibitors, also provided by Beyotime, China. The resulting cell lysates underwent centrifugation at 14,000× g for 15 minutes at a temperature of 4 °C. After centrifugation, the lysates were denatured for 10 minutes in a 5× SDS-PAGE loading buffer, again sourced from Beyotime, China. The proteins were then separated using SDS-PAGE and subsequently transferred to polyvinylidene fluoride (PVDF) membranes from Beyotime, China, for the purpose of Western blot analysis. The membranes were subjected to a blocking step using NcmBlot blocking buffer (NCM Biotech, Suzhou, China) for a duration of 10 minutes. Following this, they were incubated with primary antibodies for 8 hours at 4 °C, diluted in 5% bovine serum albumin (BSA) from Solarbio, Beijing, China. After the incubation with primary antibodies, the membranes were treated with secondary antibodies from ThermoFisher, Waltham, MA, USA, diluted in WB secondary antibody diluent solution from Beyotime, Shanghai, China, at a dilution of 1:1000 for 2 hours at room temperature. Protein detection was performed utilizing an enhanced chemiluminescence (ECL) substrate from Thermo Fisher, Waltham, MA, USA. The quantification of protein expression was conducted by evaluating the band densities of the target proteins using ImageJ software version 1.57, with the analysis based on density values relative to the GAPDH protein. The primary antibodies employed in this study included as following:

For U87 and HEB, ODC1 (ab270268, ABCAM, USA: 1:1000)
For C6 and HT-22, ODC1 (ab193338, ABCAM, USA: 1:1000)
For U87 and HEB with C6 and HT-22, GAPDH (ab181602, ABCAM, USA: 1:10000)

### 2.11 Cell proliferation assays

Cells in the logarithmic growth phase were digested with trypsin, counted, and seeded into a 96-well plate at a density of 3000 cells per well (n=6). After incubation at specific time points (24, 48, 72, and 96 hours), 10 µL of CCK-8 reagent was added to each well, and the culture plate was incubated for an additional 2 hours. The absorbance value was measured at a wavelength of 450 nm using a microplate reader. The cell proliferation rate was calculated using the formula (A_d − A_blank_d)/(A_4h − A_blank_4h). All experiments were repeated three times to ensure the reliability of the results. To verify IC50, cells were seeded into a 96-well plate at a density of 2000 cells per well (100 µL per well) and treated with the drug (THZ-2-102-1, MCE, China) for 24 hours (37 °C, 5% CO_2_). Subsequently, 10 µL of Cell Counting Kit-8 reagent (Catalog No. C0038, Beyotime) was added to each well, taking care to avoid bubble formation. After incubating the culture plate for an additional 2 hours, the absorbance value was read at a wavelength of 450 nm using a microplate reader (EnSight, PerkinElmer, USA), and the cell viability was calculated according to the manufacturer’s instructions.

### 2.12 Statistical analysis

All statistical evaluations were executed utilizing R software alongside GraphPad Prism software. The assessment of disparities between pairs of groups was carried out using either the Student’s t-test or the Wilcoxon rank-sum test, contingent upon the distribution of the data. In instances of multiple group comparisons, one-way ANOVA was employed, succeeded by Tukey’s *post hoc* test. The relationships between gene expression levels and immune cell infiltration were investigated through Spearman correlation analysis. A two-tailed p-value of less than 0.05 was deemed statistically significant.

## 3 Results

### 3.1 Identification of gluconeogenesis/lactylation-related DEGs for GBM patients

**Figure 2:**
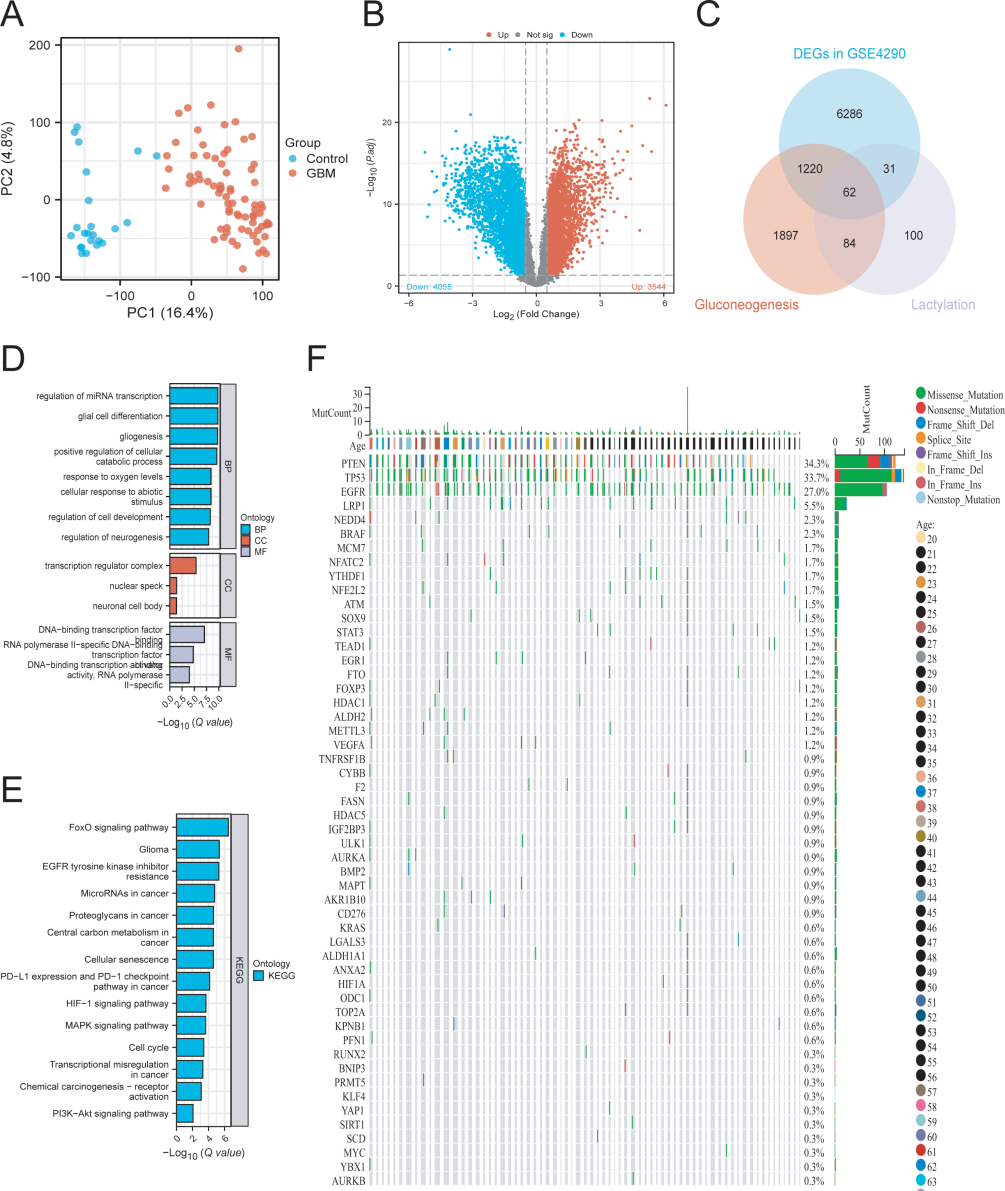
Identification of gluconeogenesis/lactylation-related DEGs for GBM patients. **(A)** PCA analysis illustration of removing batch effect results in GSE4290. **(B)** Volcano map illustration of DEGs in GSE4290. **(C)** Gluconeogenesis/lactylation-associated DEGs identification in GSE4290. **(D, E)** KEGG and GO enrichment analysis of gluconeogenesis/lactylation-associated DEGs. **(F)** Genetic variation analysis of gluconeogenesis/lactylation-associated DEGs.

After removing the batch effects of GSE4290, we identified 3544 up-regulated and 4055 down-regulated DEGs (**Figures 2A, B**). Next, DEGs were intersected with gluconeogenesis and lactylation-associated gene lists for identification of 62 gluconeogenesis/lactylation-associated DEGs (**Figure 2C**). Next, molecular functions and genetic variations of these 62 DEGs were also analyzed (**Figures 2D–F**).

### 3.2 Identification of TGL-associated shared DEGs for GBM patients

**Figure 3:**
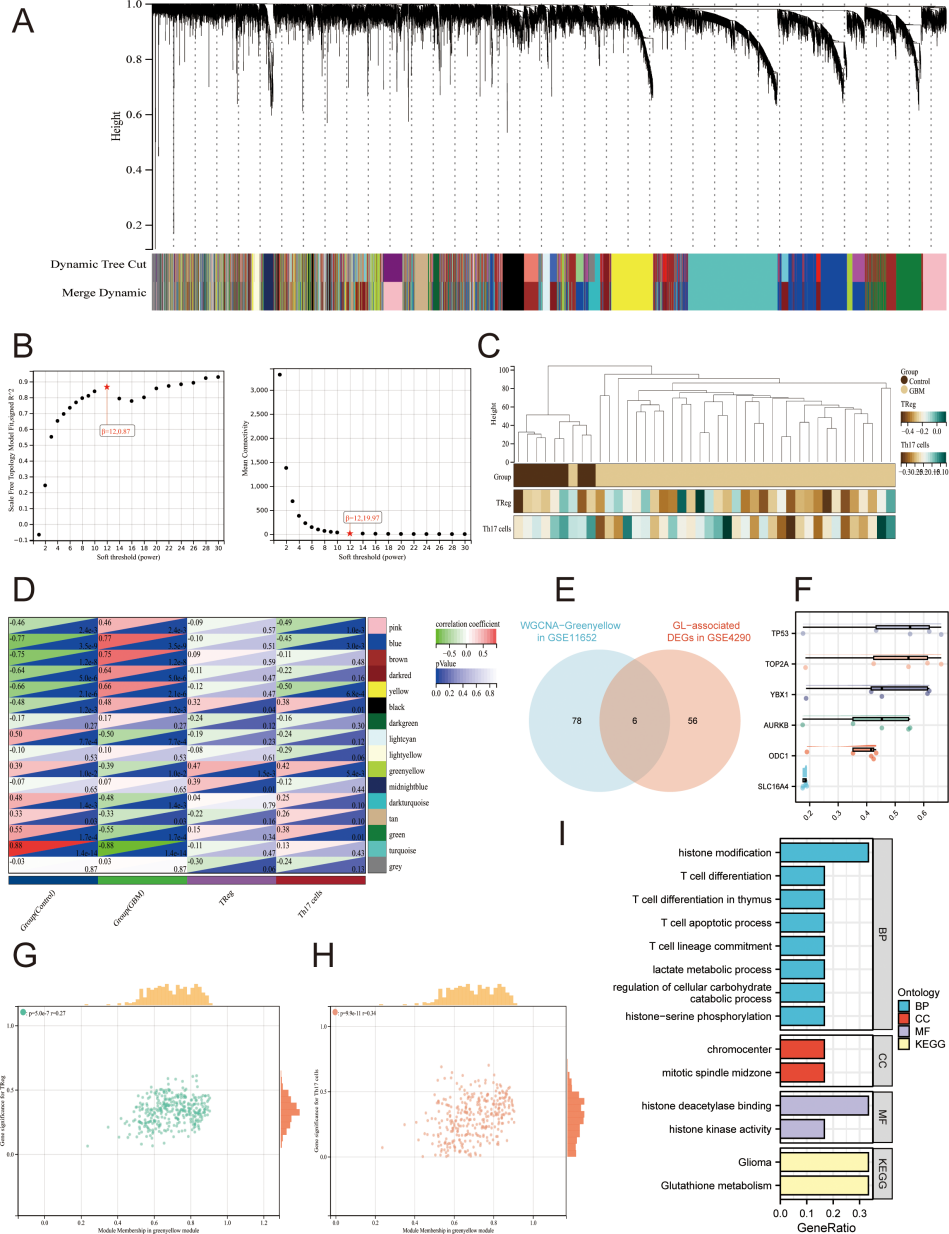
Identification of GL-associated shared DEGs for GBM patients. **(A)** Clustering tree of expression module via WGCNA analysis. **(B)** Scatterplots of representative modules of WGCNA analysis. **(C, D)** Sample clustering and Module trait relationship heatmap generated by WGCNA analysis. **(E)** GL-associated shared DEGs identification. **(F)** Friend analysis of GL-associated shared DEGs. **(G, H)** Treg/Th17 greenyellow module illustration from WGCNA analysis. **(I)** KEGG and GO enrichment analysis of 6 GL-associated DEGs.

In GBM bulk profile GSE105437, we first performed WGCNA and ssGSEA analysis for the identification of co-expression model with Treg/Th17 axis in GSE11652 and discovered that greenyellow module was the highest-correlated module associated Treg/Th17 axis (**Figures 3A–D**). Besides, we also recognized the 6 TGL-associated shared DEGs by extracted hub gene in greenyellow module and then intersected with gluconeogenesis/lactylation-associated DEGs (**Figures 3E–H**). KEGG and GO enrichment analysis indicated that these 6 TGL-associated DEGs were mainly involved in T cell regulation, histone modification, intracellular metabolism and pathogenesis of glioma (**Figure 3I**).

### 3.3 TGL-associated prognostic model construction for GBM patients

**Figure 4:**
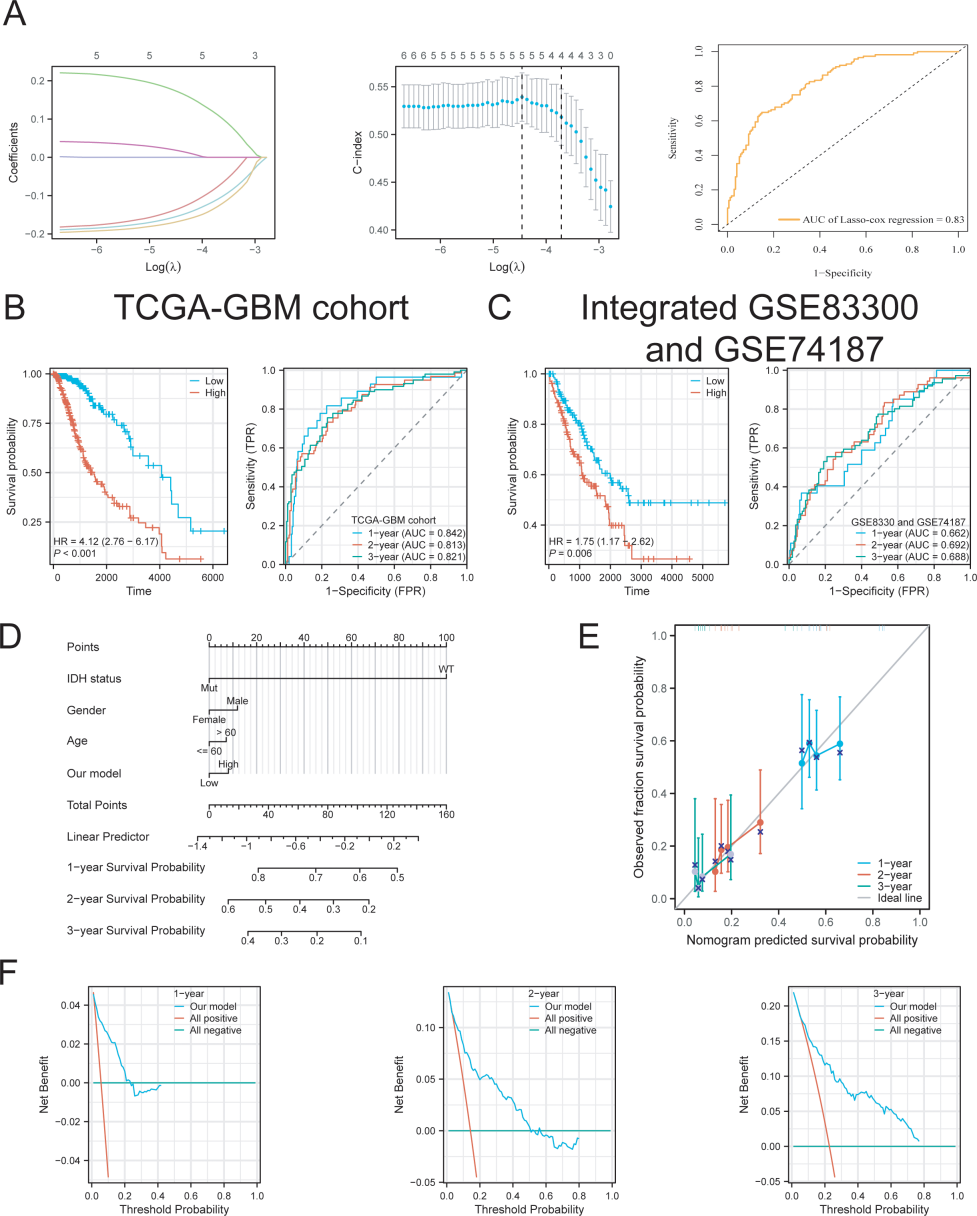
Identification of GL-associated prognostic model for GBM patients. **(A)** Lasso-cox regression analysis based on GL-associated DEGs. **(B)** GL-associated prognostic model evaluation in TCGA-GBM cohort. **(C)** GL-associated prognostic model evaluation in integrating GSE83300 and GSE74187. **(D–F)** GL-associated prognostic model efficacy nomogram examination.

We performed LASSO-Cox regression analysis based on 5 GL-related genes for construction of GL-associated prognostic model in TCGA-GBM cohort and integrated GSE83300 and GSE74187 (**Figures 4A–C**). Results indicated that the model can successfully divided patients into 2 groups and illustrated satisfied performance (**Figures 4A–C**). Next, we also validated the model efficacy in TCGA-GBM cohort, and our model illustrated the favorable accuracy and efficacy (**Figures 4D–F**).

### 3.4 TGL-associated high-risk and low-risk group immune features estimation and hub gene identification

**Figure 5:**
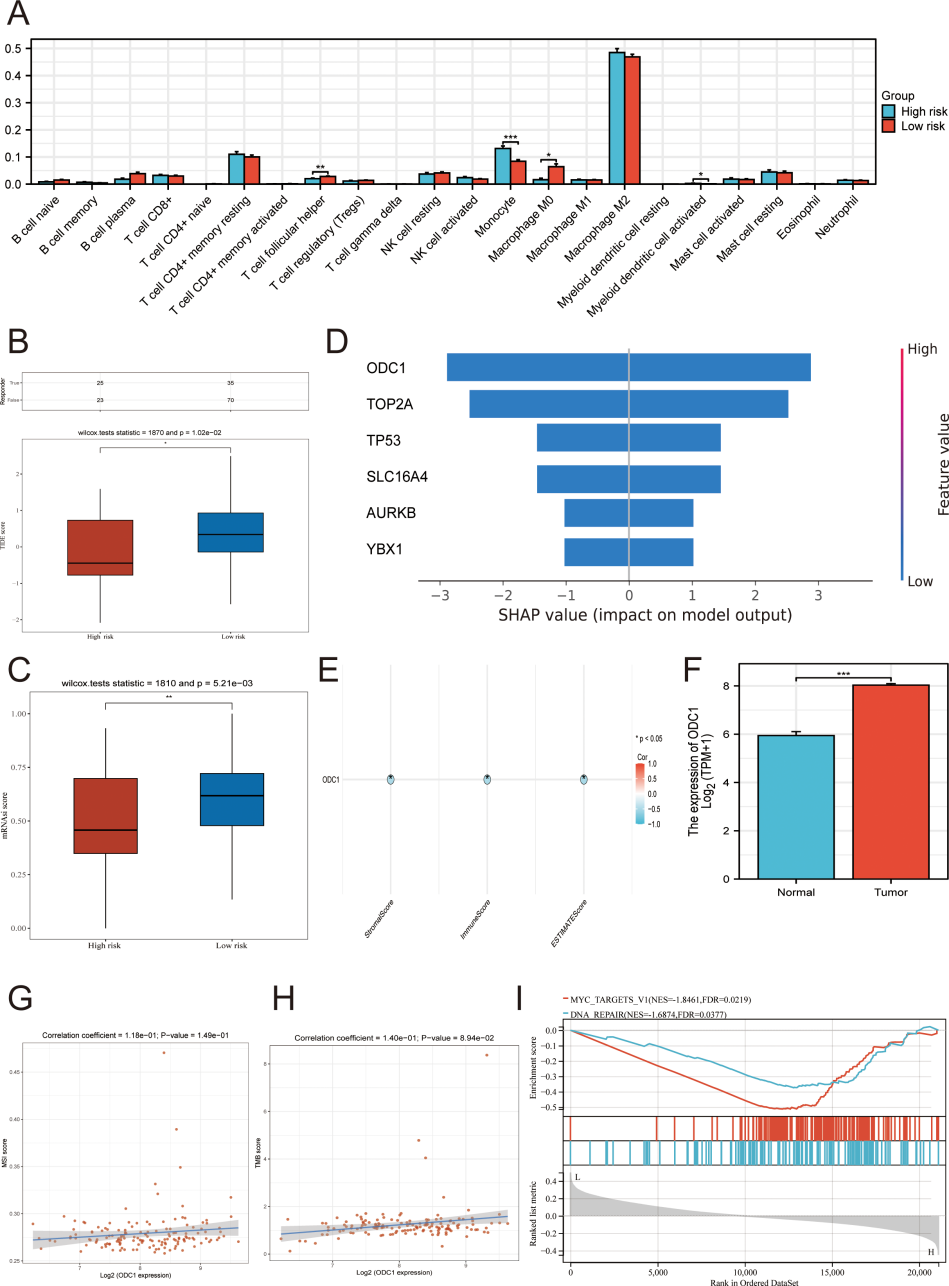
TGL-associated high-risk and low-risk group immune features estimation and hub gene identification. **(A)** Immune infiltration analysis between high-risk and low-risk groups. **(B)** TIDE analysis between high-risk and low-risk groups. **(C)** Tumor stemness between high-risk and low-risk groups. **(D)** SHAP analysis for identifying main contributor in TGL-associated prognostic model. **(E)** ESTIMATE analysis of ODC1 in TCGA-GBM cohort. **(F)** The expression analysis of ODC1 in TCGA-GBM cohort. **(G, H)** The relationship between ODC1 and MSI with TMB. **(I)** Single-gene GSEA enrichment analysis of ODC1 in TCGA-GBM cohort. *:P < 0.05, ** :P < 0.01, ***:P < 0.001.

Firstly, we compared immune cell infiltration, TIDE score and tumor stemness in high risk and low risk groups in TCGA-GBM cohort (**Figures 5A–C**). Next, SHAP analysis indicated that ODC1 can be considered as major contributor in TGL-associated prognostic model (**Figure 5D**). Besides, ODC1 was increased expression in GBM patient samples compared to normal samples in TCGA-GBM cohort (**Figure 5F**). Indeed, the relationship between ODC1 and MSI with TMB was also estimated in TCGA-GBM cohort (**Figures 5G, H**). Next, we also discovered that ODC1 was negatively associated with stromal and immune infiltration in TCGA-GBM cohort (**Figure 5E**). Besides, in TCGA-GBM cohort, we discovered that ODC1 can negatively regulate DNA repair and MYC target activity (**Figure 5I**). These results indicated that ODC1 was closely linked to GBM TME and progression.

### 3.5 TGL-associated hub gene at single-level level for GBM patients

**Figure 6:**
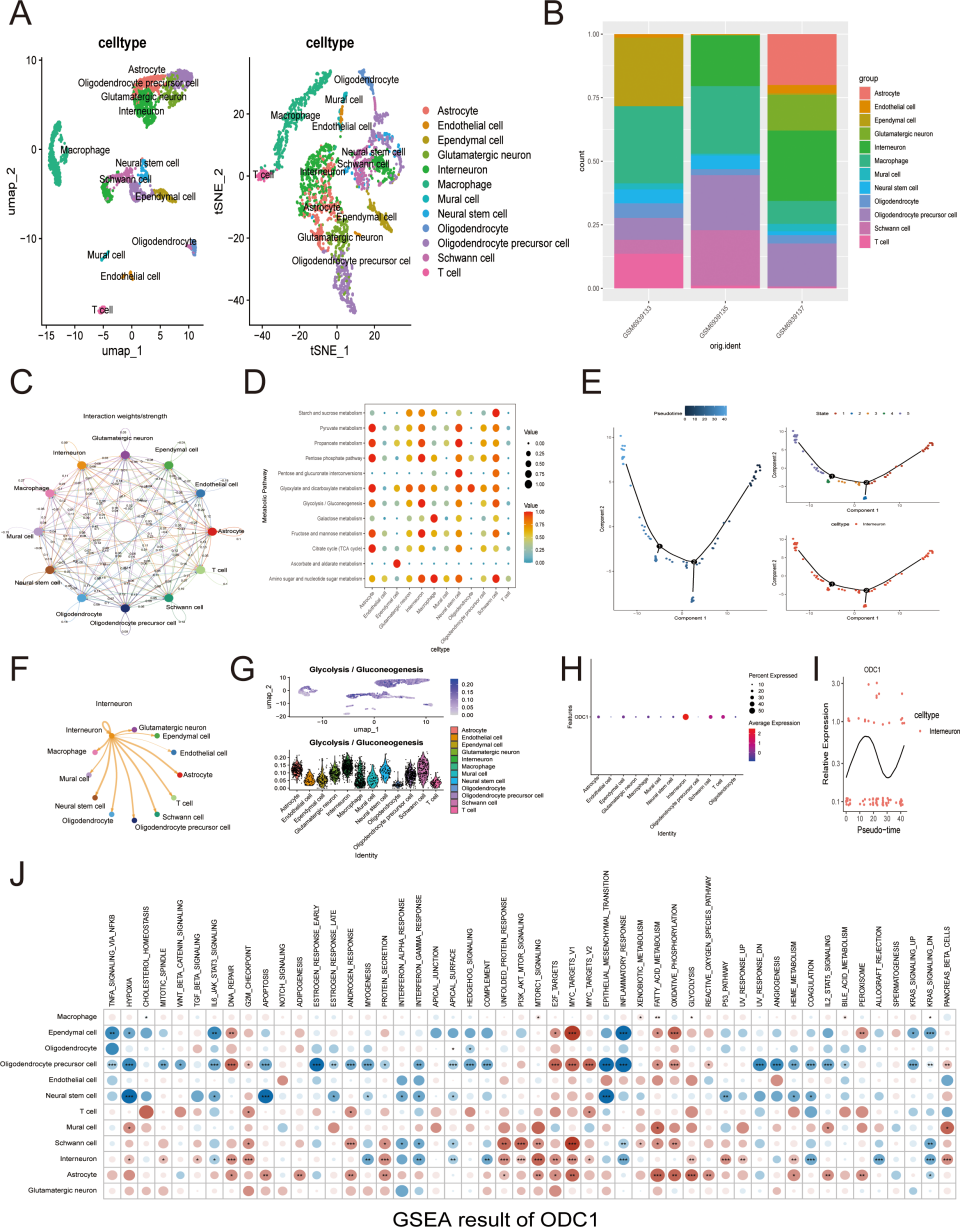
TGL-associated hub gene at single-level level for GBM patients. **(A, B)** UMAP and t-SNE illustration of annotation results. **(C, F)** Cell chat analysis among annotated cell types. **(D, G)** Energy metabolism analysis among annotated cell types. **(E, I)** Pseudo-time trajectory analysis of interneuron and temporal expression of Odc1 in interneuron. **(H)** Distribution of Odc1 among annotated 12 cell types. **(J)** Single-cell GSEA enrichment analysis of Odc1 among annotated 12 cell types. *:P < 0.05, ** :P < 0.01, ***:P < 0.001.

In GSE223063, after pre-processing of GBM single-cell dataset, we confirmed 15 cell clusters and 12 cell types (**Supplementary Figure 1A–F**; **Figures 6A, B**). Next the cell chat, energy metabolism (especially Gluconeogenesis) patterns among 12 cell types were also analyzed (**Figures 6C, D, F, G**). Next, we discovered that Odc1 was mainly distributed at interneuron and its temporal expression manner in interneuron was also estimated (**Figures 6E, H, I**). Finally. the molecular functions of Odc1 among these 12 cell types were also evaluated (**Figure 6J**).

### 3.6 ODC1-targeted therapeutic approach enrichment for GBM patients

**Figure 7:**
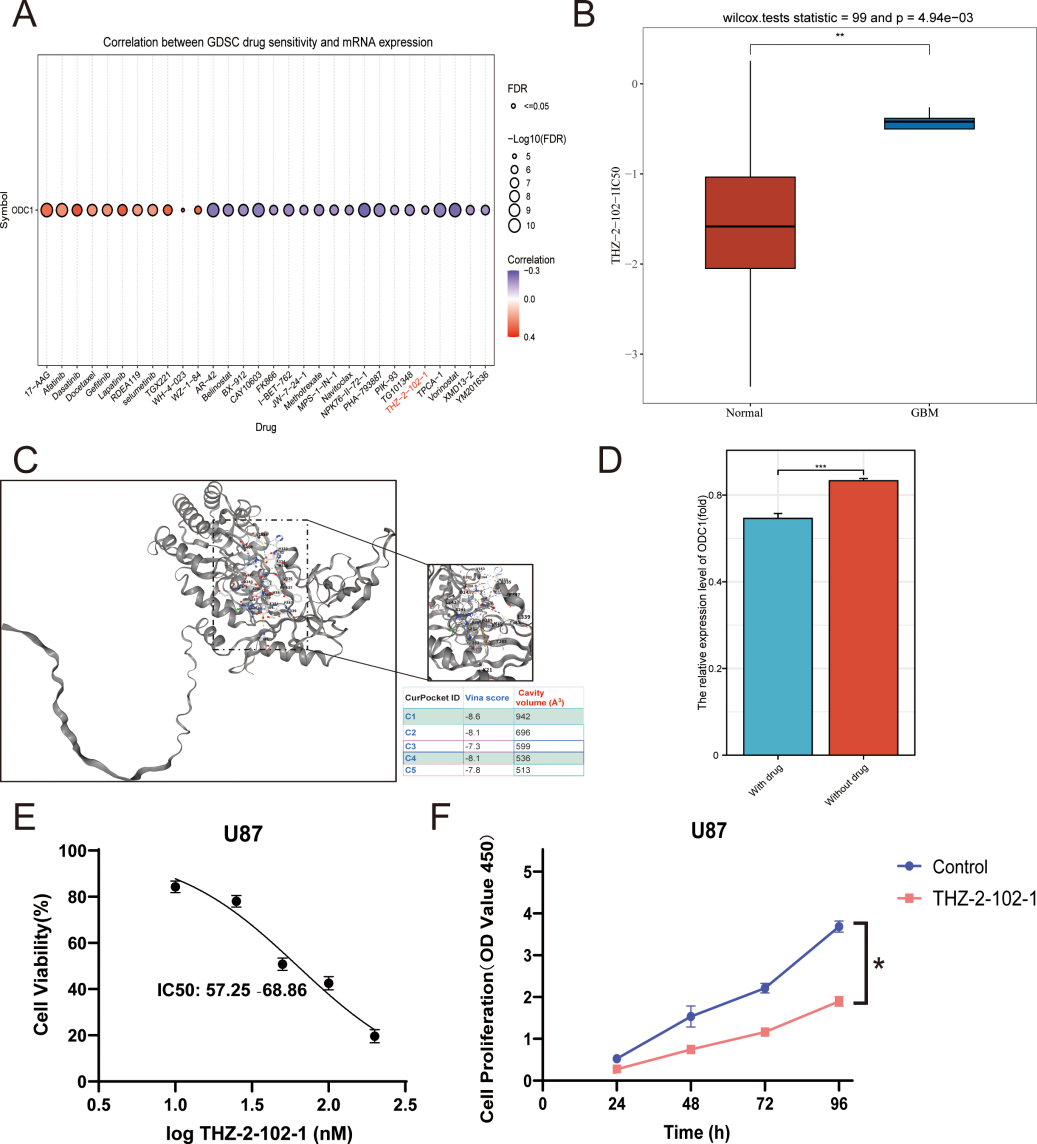
ODC1-targeted therapeutic approach enrichment for GBM patients. **(A)** GSCA database enrichment. **(B)** Ridge regression for evaluation of drug sensitivity in GBM. **(C)** Molecular docking validation. **(D)** q-RT-PCR examination of ODC1 expression. **(E)** IC50 evaluation of THZ-2-102-1. **(F)** CCK-8 evaluation of THZ-2-10–1 therapeutic effects targeting U87 cell lines. *:P < 0.05, ** :P < 0.01, ***:P < 0.001.

GSCA database confirmed the sensitivity drug targeting higher expression of ODC1, we confirmed THZ-2-102-1 (**Figure 7A**). Next, ridge regression confirmed that THZ-2-102–1 was sensitive to GBM tumor tissues (**Figure 7B**). Molecular docking illustrated the favorable binding affinity between THZ-2-102–1 and ODC1(-8.6kcal/mol) (**Figure 7C**). In U87 GBM cancer cell line, after confirming the optimal concentration of THZ-2-102-1, we discovered that THZ-2-102–1 can inhibit U87 cell growth and ODC1 expression (**Figures 7D–F**). These results indicated that THZ-2-102–1 can be considered as potential therapeutic agent for GBM treatment.

### 3.7 The association of ODC1 with TGL in GBM and GBM progression

**Figure 8:**
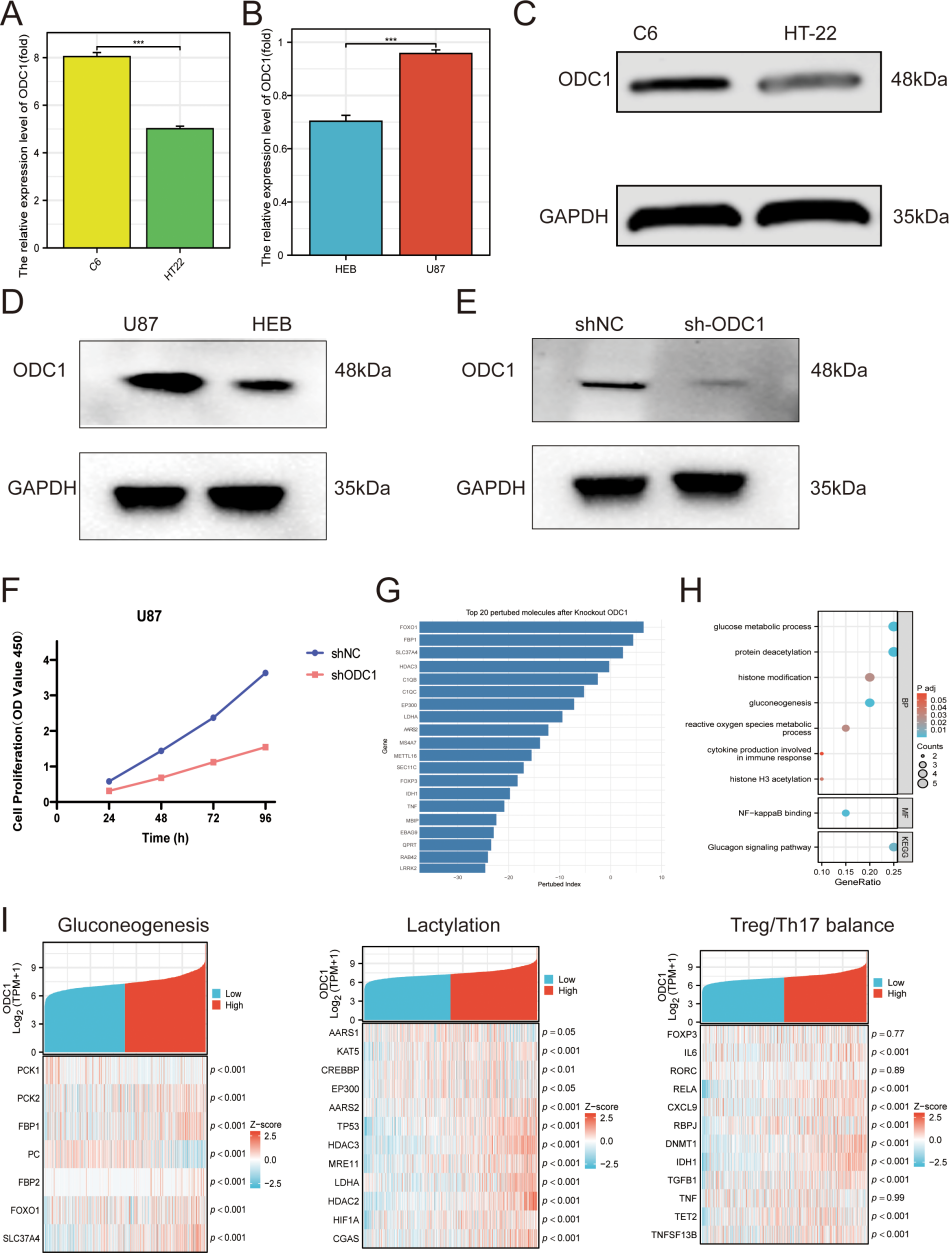
The association of ODC1 with TGL and GBM cancer cell growth. **(A–D)** Expression patterns of ODC1 in GBM human and mouse cell lines compared to normal cell lines via WB and q-RT-PCR. **(E)** WB examination of ODC1 knockdown efficacy in U87 cell lines. **(F)** CCK-8 examination. **(G, H)** Virtual KO of ODC1 in interneuron. **(I)** Co-expression pattern association of ODC1 with TGL. ***:P < 0.001.

Firstly, we performed WB and q-RT-PCR analysis for determining the expression patterns of ODC1 in human and mouse GBM cancer cell lines (C6 and U87) compared to normal control (HT-22 and HEB) (**Figures 8A–D**). Results indicated an increased pattern ODC1 in GBM cancer cell lines. Next, after knockdown of ODC1 in U87 cancer cell line, it can be witnessed that decreased cell growth patterns in U87 cancer cell line (**Figures 8E, F**). Additionally, we performed virtual KO of ODC1 in interneuron and discovered that KO of ODC1 can affect molecular and biological functions related to Treg/Th17 balance, histone modification and Glucose metabolism (**Figures 8G, H**). Next, in TCGA-GBM cohort, we discovered that ODC1 was co-expressed with Treg/Th17 axis and gluconeogenesis/lactylation key regulators (**Figure 8I**). These results indicated that ODC1 can potentially regulate Treg/Th17 axis and gluconeogenesis/lactylation in GBM and GBM cancer growth.

## 4 Conclusion and discussion

GBM remains one of the most aggressive and treatment-resistant brain tumors, posing significant challenges in clinical management and decision-making (39). Current therapeutic strategies often fall short due to the tumor’s heterogeneity and the complex tumor microenvironment, highlighting the urgent need for innovative approaches that can enhance patient outcomes (40). In this study, by employing end-to-end interpretable machine learning pipelines and multi-omics, we systematically assessed the TGL-associated predictive and therapeutic potentials for GBM patients. Besides, we also highlighted pathogenic role of ODC1 in modulation of TGL in GBM. In addition, we also discovered the therapeutic strategy (THZ-2-102-1) targeting ODC1 in GBM.

ODC1 (ornithine decarboxylase 1), an enzyme is responsible for responding growth-promoting stimuli (41). In the aspect of brain tumor, mutation of ODC1 contributes to the progression of GBM (42). Besides, ODC1 also can be considered as prognostic biomarker for pediatric medulloblastoma related to metabolic reprogramming (43). Additionally, ODC1 also can be considered as modulator for glucose and lipid metabolism (44). Metabolic reprogramming of glucose and lipid metabolism also can be considered as hallmark of GBM progression (45). Besides, an independent investigation illustrated that ODC1 was a modulator involved in gluconeogenesis in liver (46). Indeed, ODC1 can be considered as effector for excessive lactylation during metabolic reprogramming in cancer pathogenesis (44). Besides, ODC1 also can modulate CD4+T cell differentiation (47). Besides, up-regulated expressions of ODC1 modulated by HIVEP1 can modulate TH17 cell differentiation and cytokine production (48). Besides, ODC1 also can be considered as a major driver involved in immunosuppressive T cell infiltration and immune checkpoint blockade in pleural mesothelioma (49). However, previous study has not yet elucidated the role of ODC1 in co-regulation of TGL and GBM pathogenesis.

In conclusion, by employing cutting-edge machine learning pipelines and multi-omics, we first discovered and validated integrated TGL predictive and therapeutic models for GBM patients. Besides, we also first discovered ODC1 pathogenic role in GBM pathogenesis. However, there are still limitations in our study. For instance, the accuracy and efficacy of TGL-associated predictive model and therapeutic efficacy of THZ-2-102–1 should be further validated in a large and multi-center clinical cohort to enhance robustness. Besides, the molecular and immune features of ODC1 and its association with TGL in GBM and GBM progression acquired from our study were based on *in silico* screening and limited *in vitro* validation, which needs more exploration in future pre-clinical studies. Additionally, limited sample sizes of single-cell data without malignant cell annotation hinder the exact functions in GBM pathogenesis, future studies should validate ODC1 distribution and molecular characters in GBM progression in an advanced omic technology, such as spatial transcriptomic.

## Data Availability

The original contributions presented in the study are included in the article/**Supplementary Material**. Further inquiries can be directed to the corresponding author.
