# Supplementary material for: Interpretable machine learning-driven multi-omics risk stratification and drug repurposing nominates Treg/Th17 with gluconeogenesis/lactylation integration as a prognostic and druggable biomarker for glioblastoma patients
Source: Front Oncol. 2026 Jul 14;16:1761182. doi: 10.3389/fonc.2026.1761182 (PMC13407271; doi:10.3389/fonc.2026.1761182)
Supplement: Supplementary file 1 [file DataSheet1.docx]

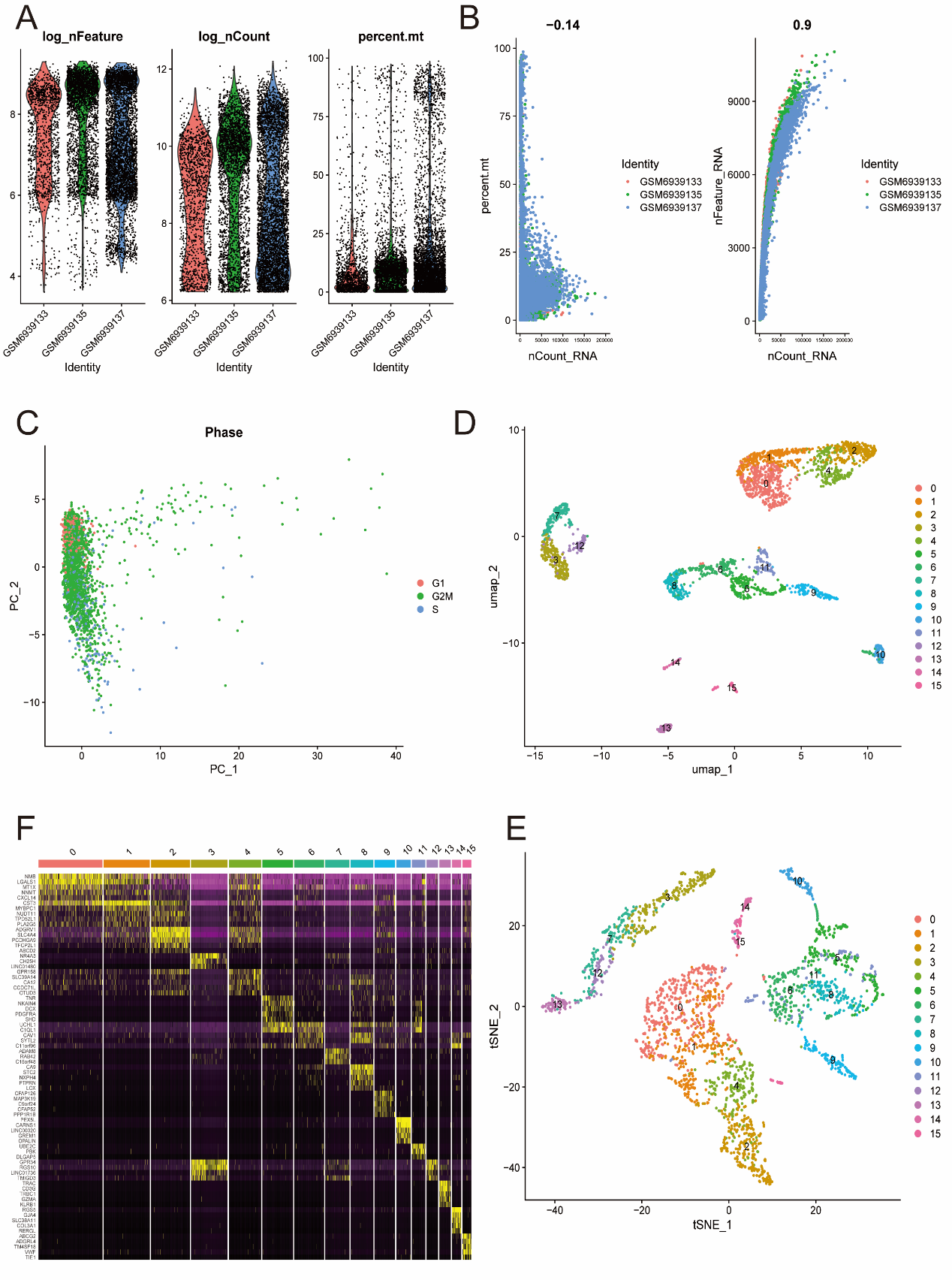


Figure S1: Pre-processing of single-cell data. **(A-B)** QC metrics of single-cell data across samples. **(C)** PCA analysis of cells based on cell cycle phase (G1, S, G2M). **(D-F)** Dimensionality reduction analysis.
